# Alicyclic Design of Sulfonated Polyimide Membranes with a Tricyclodecane Diamine for Improved Ion Crossover Blocking in Vanadium Redox Flow Batteries

**DOI:** 10.1021/acspolymersau.5c00066

**Published:** 2025-08-25

**Authors:** Chieh-Yuan Chang, Chang-Liang Liu, Shi-Jie Wang, Fu-En Szu, Hong-Yu Lin, Kao-Shu Chuang, Man-kit Leung, Yan-Cheng Lin

**Affiliations:** † Department of Chemical Engineering, 34912 National Cheng Kung University, Tainan 70101, Taiwan; ‡ Department of Chemistry, 33561 National Taiwan University, Taipei 10617, Taiwan; § Department of Green Material Technology, 83504 Green Technology Research Institute, CPC Corporation, Kaohsiung City 811, Taiwan; ∥ Advanced Research Center for Green Materials Science and Technology, National Taiwan University, Taipei 10617, Taiwan

**Keywords:** polyimide, proton exchange membrane, intrinsic porosity, vanadium permeability, rechargeable flow battery

## Abstract

Polyimides (PIs), known for high thermal stability, strength, and chemical resistance, are used in energy systems, such as fuel cells and redox flow batteries. Despite Nafion membranes offering high proton conductivity, their high cost, strong water dependency, and severe vanadium ion crossover limit their long-term stability and practical viability in vanadium redox flow batteries (VRFBs). Thus, designing high-performance proton exchange membranes (PEMs) based on PI with both selective proton conductivity and vanadium ion blocking capability has become a critical challenge. This study presents a design strategy that combines alicyclic and aromatic diamine monomers to achieve both structural and performance benefits. The rigid and bulky tricyclodecane diamine (TCDDA) is copolymerized with flexible aromatic diamines (ODA) and sulfonated diamines (BDSA) to synthesize segmented copolymers incorporated into the PI backbone. This design increases the free volume and enables controlled microphase separation for selective proton transport and vanadium blocking. To assess steric effects and chain stacking, a less bulky analogue, noborane diamine (NBDA), was also used for comparison. The TCDDA-based membranes exhibited outstanding comprehensive properties, including a tensile strength of up to 89 MPa and an elongation at break of 22.7%. Microstructural analysis revealed that TCDDA promoted orderly chain stacking and stable phase separation compared with the NBDA series, allowing for selective ion transport without the need for additional pore-forming treatments. In VRFB tests, the PEM with 10% TCDDA (T10) demonstrated an exceptionally low vanadium ion permeability (9.79 × 10^–8^ cm^2^/min), significantly outperforming Nafion and NBDA-based membranes in terms of Coulombic efficiency. Energy efficiency remains above 80% across all current densities. The T10 membrane retained its integrity and conductivity after repeated cycles, confirming excellent stability. The remarkably low vanadium ion permeability of TCDDA-based alicyclic PI further underscores its high long-term durability and selectivity.

## Graphical abstract

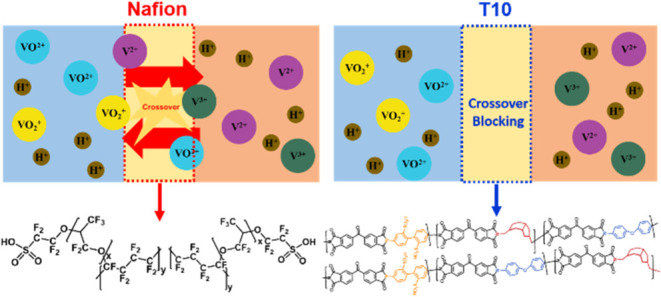

## Introduction

Proton exchange membranes (PEMs) play a pivotal role in advancing clean energy technologies, serving as key components in proton exchange membrane fuel cells (PEMFCs),^1–3^ proton exchange membrane water electrolysis (PEMWE),^4–6^ and vanadium redox flow batteries (VRFBs).^7–10^ These systems are integral to hydrogen energy production, energy storage, and the integration of renewable energy. PEMs facilitate selective proton conduction while blocking electrons and reactive species, such as gases (e.g., H_2_ and O_2_) or metal ions (e.g., vanadium ions in VRFBs), to maintain electrochemical efficiency and system stability. In VRFBs, PEMs play a critical role in balancing charges by conducting protons and preventing the crossover of vanadium ions in multiple oxidation states (V^2+^, V^3+^, VO^2+^, and VO_2_^+^). This dual functionality minimizes self-discharge, enhances Coulombic efficiency, and extends cycle life, making PEMs a crucial component for long-term energy storage applications.

Despite their significance, currently available PEM materials face critical challenges that restrict their performance and scalability. Commercially available PEM, Nafion, is predominantly utilized due to its high proton conductivity, excellent chemical durability, and good mechanical properties.^11^ However, its water dependency for proton conduction results in a sharp degradation of its performance at elevated temperatures or low humidity levels. In addition, Nafion’s phase-separated structure, hydrophilic proton-conducting channels, and hydrophobic fluorocarbon backbone enable a significant amount of crossover of both gases and ions, which is crucial in VRFB applications where vanadium ion crossover leads to electrolyte fouling and capacity loss. In addition, Nafion’s high manufacturing cost and nondegradability of its fluorinated backbone pose economic as well as environment-related concerns, making its feasibility for large-scale implementation in sustainable energy applications questionable.

To overcome these drawbacks, an investigation has been conducted on aromatic polymer-based PEMs, which offer improved thermal stability, tunable molecular architectures, and enhanced mechanical properties. Some representative aromatic polymers include sulfonated poly­(ether ether ketone) (SPEEK),^12,13^ sulfonated poly­(arylene ether) (SPAE),^14,15^ and polyimide (PI).^16–18^ Among all of them, PI is a promising candidate because it has very high thermal resistance, chemical resistance in a strong acid environment, and flexibility in structural design. PI’s rigid aromatic backbone and imide linkages ensure durability in high-temperature PEMFC and acidic VRFB applications. With the incorporation of proton-conducting functional groups like sulfonic acid (−SO_3_H), phosphonic acid (−PO_3_H_2_), or imidazole, PI membranes retain proton conductivity under hot and dry conditions, lower swelling, and higher, denser morphologies than Nafion. All of these attributes provide better resistance to gas and ion crossover, making PI-based PEMs well-suited for VRFB applications. In addition, PI can be synthesized from low-cost, nonfluorinated raw materials, thereby reducing manufacturing costs and aligning with the principle of environmentally friendly material development.

To effectively enhance the performance of PI-based PEMs in VRFBs, various structural design strategies for sulfonated polyimide (SPI) membranes have been proposed.^19–21^ Wang et al. introduced acid–base pair structures by adjusting the ratio of sulfonic acid and imidazole groups, thereby forming continuous proton transport channels while effectively suppressing vanadium ion crossover.^10^ Li et al. synthesized branched and covalently self-cross-linked SPI membranes using an X-shaped imidazole-functionalized tetramine, achieving significantly improved ion selectivity and cycling stability compared to Nafion.^22^ Xu et al. employed β-cyclodextrin as a sacrificial template to create porous cross-linked PI membranes, where the resulting interconnected network enhanced ionic conductivity and long-term durability, sustaining over 2,250 stable charge–discharge cycles.^23^ Chu et al. adopted a one-pot polymerization approach to fabricate microporous SPI membranes with a gradient distribution of sulfonic acid groups, enabling fine control of pore size to balance proton transport and vanadium ion blocking, resulting in energy efficiencies comparable to commercial Nafion membranes.^24^ In summary, these studies demonstrate the effectiveness of molecular architecture and microstructural control in advancing high-performance and durable membranes for next-generation VRFBs.

There has been no report about using alicyclic monomers in SPIs for creating intrinsic porosity while maintaining structural rigidity. Therefore, this research utilizes the alicyclic monomer tricyclodecane diamine (TCDDA) in PI-based PEMs to improve inherent microporosity, free volume, and structural integrity.^25^ TCDDA’s specific molecular geometry, with its steric hindrance, facilitates the creation of proton-conducting channels and vanadium ion-blocking barriers without the incorporation of any additional pore-forming treatment. This spatial effect of alicyclic monomers is conducive to enhancing the PEM performance across VRFB applications, where selective ion conduction is crucial. TCDDA is produced from a petrochemical byproduct of dicyclopentadiene (DCPD) through a catalytic addition process. The value-added transformation of DCPD is a critical issue for developing low-carbon emission chemicals. By utilizing byproduct streams instead of virgin petrochemical feedstocks, the production of TCDDA significantly reduces the reliance on primary raw materials. It helps lower carbon emissions compared with traditional materials, such as Nafion. Moreover, TCDDA’s alicyclic nature promotes specific aggregation behavior that enhances membrane robustness and electrochemical stability. To study the systematic influence of alicyclic skeletons on the performance of the membrane, in this study, PI-based PEMs made from TCDDA are compared with PEMs made from less bulky norbornane diamine (NBDA), as well as a control group without the addition of any alicyclic component. All of the membranes were prepared through chemical modification by introducing sulfonic acid functional groups to facilitate proton conductivity. The structure, morphology, and electrochemical properties were thoroughly characterized using sophisticated methods, including grazing-incidence wide-angle X-ray scattering (GIWAXS), grazing-incidence small-angle X-ray scattering (GISAXS), scanning electron microscopy (SEM), atomic force microscopy (AFM), and electrochemical impedance spectroscopy (EIS). The development of TCDDA-based SPI membranes represents a crucial step toward achieving high-performance and sustainable PEMs for next-generation energy applications. By leveraging the environmentally friendly nature and structural benefits of TCDDA, this method circumvents the economic and ecological constraints of conventional fluorinated membranes.

## Experimental Section

### Materials

Dimethyl sulfoxide (DMSO) was obtained from UniRegion. 3,3′,4,4′-Benzophenonetetracarboxylic dianhydride (>97%, BTDA) and triethylamine (>98%, TEA) were purchased from Thermo Scientific. 2,2′-Benzidinedisulfonic acid (>70%, BDSA) and 4,4′-diaminodiphenyl ether (>98%, ODA) were purchased from Tokyo Chemical Industry Company (Tokyo, Japan). BDSA was first dissolved in ethanol and subsequently neutralized with triethylamine at 60 °C to obtain its triethylammonium salt form (BDSA-Et_3_NH), as illustrated in Scheme S1.^26^ Tricyclodecane diamine (TCDDA) was synthesized according to a reported method.^25,27,28^ Bis­(aminomethyl)­norbornane (>98%, NBDA) was obtained from MACKLIN. The dianhydride was dried in a vacuum oven at 150 °C for 24 h before use.

### Synthesis of the Aromatic SPIs

To synthesize PI materials with a block copolymer structure, we designed a reaction scheme involving two prepolymer segments of distinct compositions. These segments were coupled via end-group condensation to form the final block copolymer backbone. As shown in Scheme 1, in the first formulation, the sulfonated monomer BDSA-Et_3_NH (2.0 mmol) and BTDA dianhydride (2.2 mmol) were dissolved in 10 mL of DMSO at a solid content of 15–20 wt %, resulting in a PAA segment terminated with anhydride end groups. In the second formulation, the ODA (2.0 mmol) and BTDA (1.8 mmol) were reacted under the same conditions to obtain a PAA segment with amine end groups. Both prepolymers were stirred separately at room temperature for 6 h and then combined for a further 18 h of reaction to facilitate end-group condensation and formation of the block copolymer chain. After polymerization, the viscous PAA solution was precipitated in ethanol to yield a pale yellow solid. The precipitate was collected by vacuum filtration, thoroughly washed with methanol, and dried under vacuum to obtain a solid PAA powder. The resulting PAA solution was cast onto a clean glass substrate using a 500 μm gap film applicator to prepare the final PEM. Thermal imidization was carried out in a vacuum oven via stepwise heating: 40 °C for 6 h, 100 °C for 2 h, 200 °C for 2 h, and 270 °C for 2 h, with a controlled heating rate of 1.5 °C/min. After it was cooled to room temperature, the fully imidized film was peeled from the glass substrate and stored in a desiccator for subsequent characterization.

**Scheme 1:**
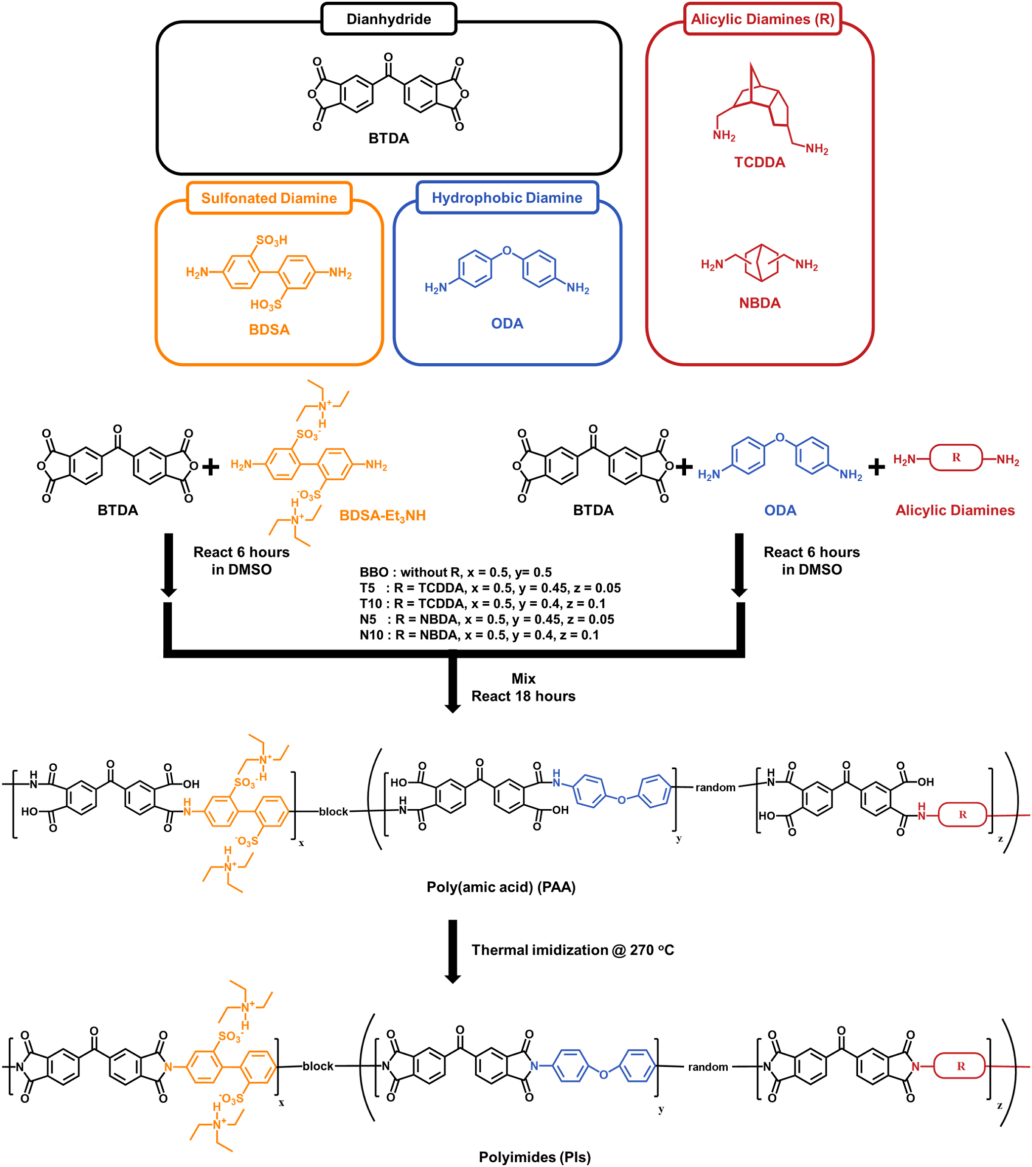
Synthesis of the SPIs and the Chemical Structures of the Constituent Monomers

### Synthesis of the Alicyclic SPIs

The synthesis of the alicyclic block copolymer followed a procedure similar to that of the aromatic SPIs, with the primary difference being the partial substitution of ODA in the amine-terminated polymer segment with two different alicyclic diamines—TCDDA and NBDA, at 5 or 10 mol % relative to the total diamine content. Taking the T5 formulation as an example, the synthesis was divided into two parts. In the first part, BDSA (2.0 mmol) was dissolved in 10 mL of DMSO, and the mixture was stirred thoroughly. BTDA (2.2 mmol) was then slowly added to form an anhydride-terminated prepolymer. In the second part, ODA (1.8 mmol) and TCDDA (0.2 mmol) were dissolved in another 10 mL of DMSO with the addition of acetic acid (0.44 mmol), followed by the slow addition of BTDA (1.8 mmol) to generate an amine-terminated prepolymer. Both solutions were stirred separately at room temperature for 6 h, combined, and reacted for an additional 18 h to complete the chain extension, yielding a PAA block copolymer. After purification and solution preparation, the resulting PAA solution was cast onto a clean glass substrate and thermally imidized under the same conditions. After being cooled to room temperature, the fully imidized alicyclic SPI film was carefully peeled off the glass and stored in a desiccator for further analysis.

### Chemical Structure Characterizations

Nuclear magnetic resonance (NMR) spectra were recorded using a BRUKER AV-500 spectrometer operating at 500 MHz, with deuterated dimethyl sulfoxide (d-DMSO) as the solvent. Fourier-transform infrared spectroscopy (FTIR) was performed by using a Thermo Scientific Nicolet 6700 spectrometer equipped with an attenuated total reflectance (ATR) accessory. The inherent viscosity (η_inh_) of the PAA samples was measured at 30 °C using an Ostwald viscometer and calculated according to the following equation $\eta_{\mathrm{inh}}=\frac{\ln\left(\eta_{r}\right)}{c}$ , where η_r_ = η/η_0_, *c* is the polymer concentration (0.5 g dL^–1^, in DMSO) and η and η_0_ are the viscosities of the polymer solution and pure DMSO, respectively. Number-average molecular weight (*M*_n_) and dispersity (*Đ*) were determined by gel permeation chromatography (GPC) using a Schambeck SFD GmbH system equipped with a refractive index (RI) detector. Separation was performed on a TSK gel MultiporeH XL-M polystyrene column with *N*,*N*-dimethylformamide (DMF) as the mobile phase at a flow rate of 1.0 mL/min and a column temperature of 40 °C. Calibration was done using polystyrene standards to evaluate polymerization efficiency and molecular weight distribution.

### Physical Characterizations of the Membranes

The film density (ρ) was measured at room temperature by using a SHIMADZU UW420H solid density analyzer with deionized water as the testing medium. Thermal properties were assessed by thermogravimetric analysis (TGA) using a TA Instruments TGA50 instrument under a nitrogen atmosphere at a heating rate of 10 °C/min to determine the thermal decomposition temperature (*T*_d_^5%^). Glass transition temperature (*T*_g_) was determined using a TA Instruments DSC25 under the same heating conditions. Thermomechanical properties, including the coefficient of thermal expansion (CTE), were analyzed by using a TA Instruments Q400 thermomechanical analyzer (TMA) from room temperature to 250 °C under air. The CTE was calculated over the temperature range 50–150 °C at a heating rate of 5 °C/min. Mechanical properties, including ultimate tensile strength (UTS) and elongation at break (ε_b_), were measured using a Shimadzu EZ-Test universal testing machine at a stretch rate of 3 mm/min. Results represent the average of the three replicates.

Film surface morphology was characterized by using a Hitachi SU8230 scanning electron microscope (SEM). The thermally imidized SPI films were mounted on conductive adhesive tape and sputter-coated with a thin layer of gold to enhance the conductivity and imaging quality. Observations were conducted under high vacuum at an accelerating voltage of 10 kV to examine the surface microstructure and molecular ordering and to evaluate the impact of molecular design on surface uniformity and microporous structure formation. GIWAXS and GISAXS were performed at the TLS BL23A1 and TPS BL25A1 beamlines of the National Synchrotron Radiation Research Center (NSRRC) in Taiwan. The SPI films, with thicknesses of approximately 4 μm, were prepared by spin-coating PAA solutions onto silicon wafers, followed by thermal imidization; the incident angles were fixed at 0.2 and 0.05°, and the X-ray wavelengths were 1.24 and 0.827 Å, respectively. The *d*-spacing was calculated using the following equation $d=\frac{2\pi }{q}$ , where *q* is the scattering vector obtained from one-dimensional integration of the two-dimensional (2D) patterns. The water uptake (WU) of the PI films was determined based on the relative weight gain after immersion in water, providing insight into the membrane’s hydrophilicity and water retention capacity. Meanwhile, the swelling behavior was evaluated by measuring the dimensional change in both the in-plane and through-thickness directions, offering a quantitative view of the volume expansion. To capture the anisotropic nature of swelling, the swelling ratio (SR) was used, with SR_*xy*_ and SR_*z*_ describing the in-plane (*xy*) and through-thickness (*z*) swelling ratios. The corresponding calculation equations are as follows

1 $$\mathrm{WU}=\frac{\left(W-W_{0}\right)}{W_{0}}$$

2 $$\mathrm{SR}=\frac{\left(L-L_{0}\right)}{L_{0}}$$

where *W* is the weight of the wet film, *W*_0_ is the weight of the dry film, *L* is the length after water absorption, and *L*_0_ is the original dry length.

### Ion-Exchange Characterizations of the Membranes

In the ion-exchange capacity (IEC) test, the dried membrane was cut into appropriate dimensions, and its dry weight was recorded. The sample was then immersed in a 1 M HCl solution for 24 h to ensure that all exchangeable functional groups were converted to the H^+^ form. After rinsing thoroughly with deionized water, the membrane was soaked in 30 mL of 1 M NaCl solution for another 24 h to allow ion exchange between the H^+^ ions in the membrane and Na^+^ ions in the solution. Finally, the concentration of H^+^ released into the NaCl solution is determined via titration, and the IEC value of the SPI membrane could be calculated using the following equation

3 $$\mathrm{IEC}=\frac{C_{\mathrm{NaOH}}V_{\mathrm{NaOH}}}{W_{0}}$$

where *V*_NaOH_ and *C*_NaOH_ represent the consumed volume and molar concentration of NaOH solution, respectively, and *W*0 is the dry membranes’ weight. Proton conductivity (σ) of the membranes was measured by using electrochemical impedance spectroscopy (EIS). A custom H-cell setup was employed in which the membrane was placed between two compartments filled with the 1 M H_2_SO_4_ electrolyte solution, using platinum electrodes. Impedance measurements were conducted using a CHI6273E multichannel electrochemical workstation (CH Instruments, Inc.) over the frequency range of 1 MHz to 0.1 Hz with a 10 mV AC perturbation. The high-frequency intercept of the Nyquist plot provided the resistance (*R*), and σ was calculated using the equation

4 $$\sigma =\frac{L}{\left(R\times A\right)}$$

where *L* is the membrane thickness and *A* is the effective area.

Vanadium ion permeability was assessed using a H-cell setup, where the membrane was clamped between two half-cells. The left half-cell contained 1 M VOSO_4_ and 2 M H_2_SO_4_ as the vanadium ion source, while the right half-cell contained 1 M MgSO_4_ and 2 M H_2_SO_4_ as the receiving solution. To maintain concentration uniformity, both solutions were continuously stirred. Samples were periodically collected from the right half-cell and analyzed using ultraviolet–visible (UV–Vis) spectroscopy to monitor the concentration of VO^2+^ over time. Notably, VO^2+^ (vanadium­(IV) ion) exhibits a distinct absorption peak at 765 nm, which served as the basis for quantitative analysis.^29^ The vanadium ion permeability (*P*) was calculated according to the following equation^30^

5 $$P=\frac{L\times V_{R}}{A\times \left(C_{0}-C_{t}\right)}\times \frac{dC}{dt}$$

where *L* and *A* represent the membrane thickness and effective area, respectively; *V*_R_ is the volume of the receiving solution in the right half-cell; *C*_0_ and *C*_t_ are the VO^2+^ concentrations in the left and right half-cells at time *t*, respectively; and σ denotes the proton conductivity of the membrane, which reflects its ion transport performance.

### Rechargeable Flow Battery Characterizations

In the vanadium redox flow battery (VRFB) test, T5, T10, and commercial Nafion 212 membranes were selected for comparative evaluation of their performance in VRFBs. The membranes, with an effective area of 10 cm^2^, were sandwiched between carbon felt electrodes (5 mm thick) and assembled into a single-cell test station. For the electrolytes, the positive half-cell contained 100 mL of a 1.6 M VO^2+^/VO_2_^+^ solution in 4.2 M H_2_SO_4_, while the negative half-cell was filled with 100 mL of a V^2+^/V^3+^ solution at the same concentration of sulfuric acid. Charge–discharge cycling was conducted at a constant voltage of 50 mV under continuous flow conditions, with current densities ranging from 40 to 80 mA/cm^2^, using a battery tester. Before the test was started, nitrogen gas was used to purge the electrolyte reservoirs to prevent oxidation of V^2+^. During the activation stage, VO^2+^ in the positive half-cell was reduced to V^3+^ through charging, while VO_2_^+^ in the negative half-cell was oxidized to V^5+^. As the battery was operated, an additional VO^2+^ solution was supplied to the positive side once sufficient V^5+^ was generated, ensuring ongoing redox reactions. Concurrently, V^3+^ in the positive half-cell was further reduced to V^2+^, and VO_2_^+^ in the opposing side was oxidized again to V^5+^. This design helped to maintain the chemical stability of the electrolyte throughout the testing process. Coulombic efficiency (CE), voltage efficiency (VE), and energy efficiency (EE) under different current densities were calculated according to the specified equations to systematically evaluate the overall membrane performance in VRFB applications.^31^

6 $$\mathrm{CE}=\frac{\mathrm{discharge capacity}}{\mathrm{charge capacity}}\times 100\%$$

7 $$\mathrm{VE}=\frac{\mathrm{discharge voltage}}{\mathrm{charge voltage}}\times 100\%$$

8 EE=CE×VE

## Results and Discussion

### Synthesis of the SPIs Studied

In this study, BTDA was selected as the dianhydride monomer to impart excellent rigidity and thermal stability to the polymer, serving as a stable backbone core. For the aromatic diamines, ODA with a flexible ether linkage and BDSA with sulfonic acid groups were copolymerized to construct a backbone structure with good film-forming ability, chemical stability, and intrinsic proton conductivity. This molecular design strategy emphasizes the use of hydrophilic–hydrophobic segments to induce microphase separation within the polymer matrix, thereby creating well-defined ionic pathways and structural domains. The chemical structures and functional characteristics of each monomer are illustrated in Scheme 1. To further investigate the effects of steric hindrance and chain stacking distance on membrane microstructure and ion transport behavior, two alicyclic diamines with different spatial effects, TCDDA and NBDA, were introduced to replace the ODA partially. TCDDA has a rigid polycyclic structure with substantial steric hindrance, which helps disrupt chain stacking and improve the ion transport selectivity. NBDA, with a more moderate steric hindrance, provides a helpful comparison between flexible ODA and the bulky TCDDA. The incorporation of alicyclic diamines (TCDDA and NBDA) enhances chain distortion and spatial rigidity, which improves the mechanical integrity and ionic selectivity of the resulting membranes. All polymers were synthesized by using a two-step method. First, BTDA was reacted with sulfonated diamine BDSA and either ODA or alicyclic diamines (TCDDA or NBDA) to prepare PAA precursors with anhydride and amine terminal groups. These were then mixed to form end-capped PAAs, enhancing their structural stability. The final SPIs were obtained through a stepwise thermal imidization process. TCDDA and NBDA, being alicyclic diamines with high basicity, tend to form tight salts with dianhydrides during the reaction; their bulky three-dimensional (3D) structures and significant steric hindrance facilitate the formation of stable salt complexes when reacting with aromatic dianhydrides, leading to precursor precipitation and decreased reaction stability. This disrupts the stoichiometric balance, resulting in nonequivalent polymerization, which suppresses the polymerization process and lowers the molecular weight of the final products. To effectively suppress salt formation and maintain reaction stability, a small amount of acetic acid was added to neutralize the strong basicity of the alicyclic diamines.^32,33^ Accordingly, acetic acid was introduced into the reaction system as a cosolvent and buffering agent to stabilize the reaction and improve solubility. The membranes were fabricated by using a solution casting method, followed by solvent removal and thermal imidization to form structurally stable PI films. This study compares three structural design strategies, aromatic (BBO), NBDA-based (N5 and N10), and TCDDA-based (T5 and T10) membranes, and systematically investigates the influence of sulfonic acid aggregation, backbone rigidity, and chain stacking distance on proton conductivity, ion selectivity, and mechanical stability. These insights help elucidate the structure–property relationships of SPI membranes for VRFB applications.

### Chemical Structure Characterizations of the SPIs Studied

To verify the chemical structure of the synthesized polymers, ^1^H NMR spectroscopy was investigated, as shown in Figure 1. By comparing the integral ratios of main-chain protons to those of the alicyclic diamines, the conversion efficiency of these diamine units was semiquantitatively evaluated. The actual contents of TCDDA in samples T5 and T10 were 4.67 and 8.67 mol %, respectively. In contrast, the NBDA contents in N5 and N10 were 3.01 and 6.58 mol %, respectively, primarily due to the presence of structural isomers in the NBDA monomer. These isomers introduce significant steric hindrance during polymerization, limiting the accessibility and reactivity of the functional groups involved. To evaluate the molecular weight characteristics, the SEC results are shown in Figure S1a. The molecular weights showed that the resulting polymers had *M*_n_ ranging from 29,800 to 44,700. The η_inh_ measurements fell within the range of 0.43–0.65 dL/g, supporting the conclusion that the synthesized polymers possessed sufficient molecular weight and chain entanglement for good film-forming capability. Both the TCDDA and NBDA series employed acetic acid during synthesis to neutralize ammonium salt byproducts and minimize their interference with the polymerization process. Nevertheless, even under identical conditions, NBDA-based polymers exhibited lower molecular weights. This difference is attributed to the molecular structure of NBDA, which contains structural isomers that result in an uneven distribution of reactive sites and increased steric hindrance. These factors not only reduce the overall reactivity but also lead to nonstoichiometric polymerization, ultimately limiting chain growth and the development of final molecular weight. Next, the degree of imidization and structural integrity of the resulting polymers were further confirmed by FTIR analysis, as shown in Figure S1b. All samples exhibited characteristic imide absorptions at 1770 and 1710 cm^–1^, corresponding to the symmetric and asymmetric stretching vibrations of the imide carbonyl groups. A strong absorption at 1376 cm^–1^ was also observed, representing the C–N stretching vibration within the imide ring. These spectral features confirm the successful conversion of PAA to PI structures and formation of the SPI backbone. Overall, the results from NMR, GPC, and FTIR analyses verify the structural fidelity and successful synthesis of the materials, offering a reliable basis for subsequent property evaluations and practical applications.^34^

**Figure 1:**
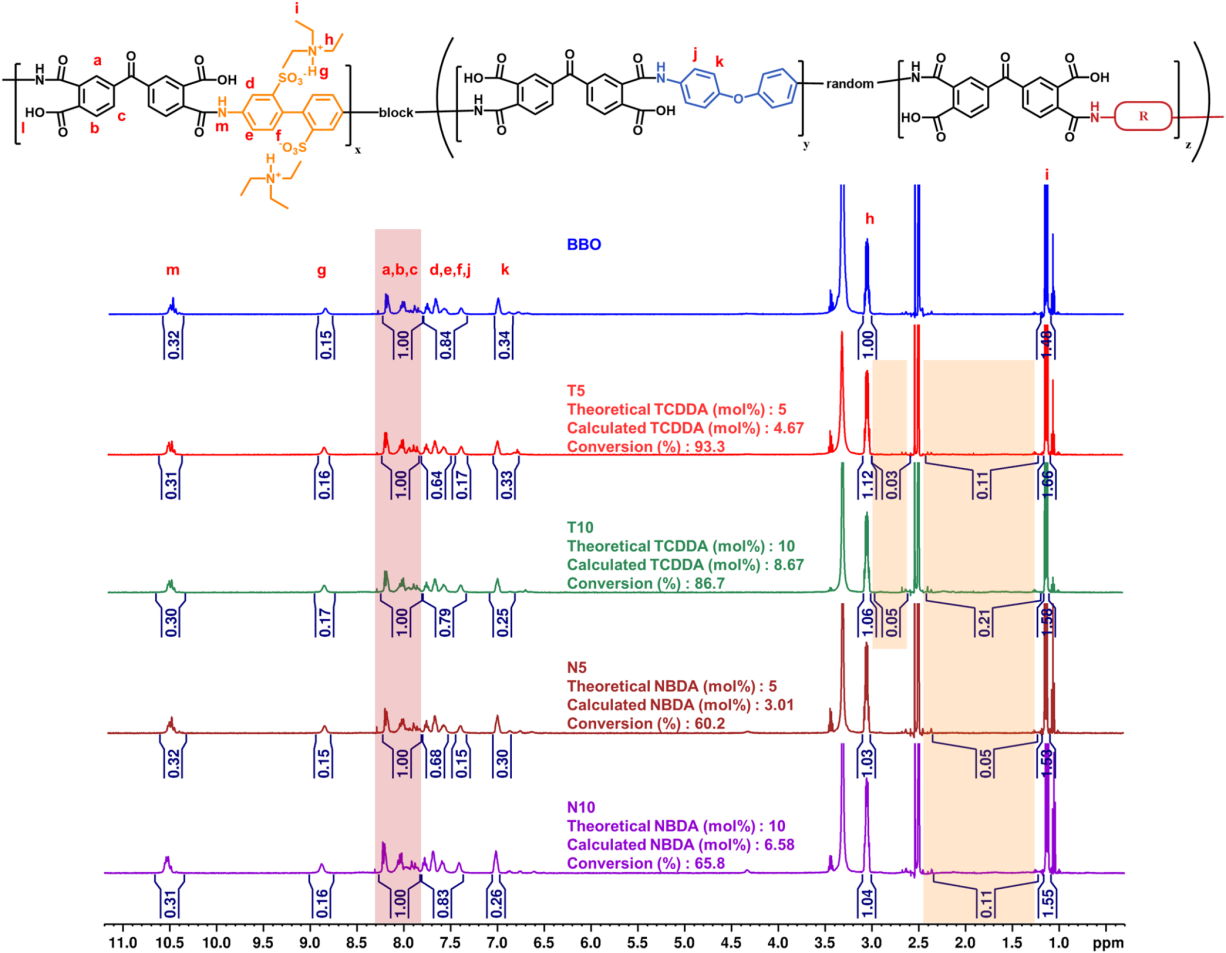
^1^H NMR spectra of the PAA precursors in *d*-DMSO.

### Thermal and Mechanical Properties of the SPIs Studied

After the structures of PAA and SPI were confirmed, the thermal stability of the synthesized SPI films was evaluated by TGA, with the results shown in Figure 2a. All samples exhibited excellent thermal stability with *T*_d_^5%^ ranging from 328 to 351 °C. A distinct two-step thermal decomposition behavior was observed. The first stage of weight loss occurred between 300 and 500 °C, which is attributed to the decomposition of sulfonic acid groups (−SO_3_H). In this stage, samples containing alicyclic diamines exhibited a more pronounced weight loss, particularly the N10 film. This is likely due to the high content of NBDA, which may induce local structural irregularities or phase separation, thereby compromising the thermal stability. The second stage of decomposition began at 500 °C and was extended up to 650 °C, corresponding to the thermal degradation of the SPI backbone. This is consistent with the reported degradation temperatures of conventional aromatic PI chains in the literature. Notably, the T10 film showed the slowest mass loss throughout the entire temperature range, indicating superior thermal stability. This performance is attributed to the high rigidity and steric hindrance introduced by TCDDA, which restricts segmental mobility and enhances thermal resistance. Overall, the incorporation of a moderate amount of alicyclic diamine—particularly TCDDA—can effectively enhance the thermal durability and structural integrity of SPI films.

**Figure 2:**
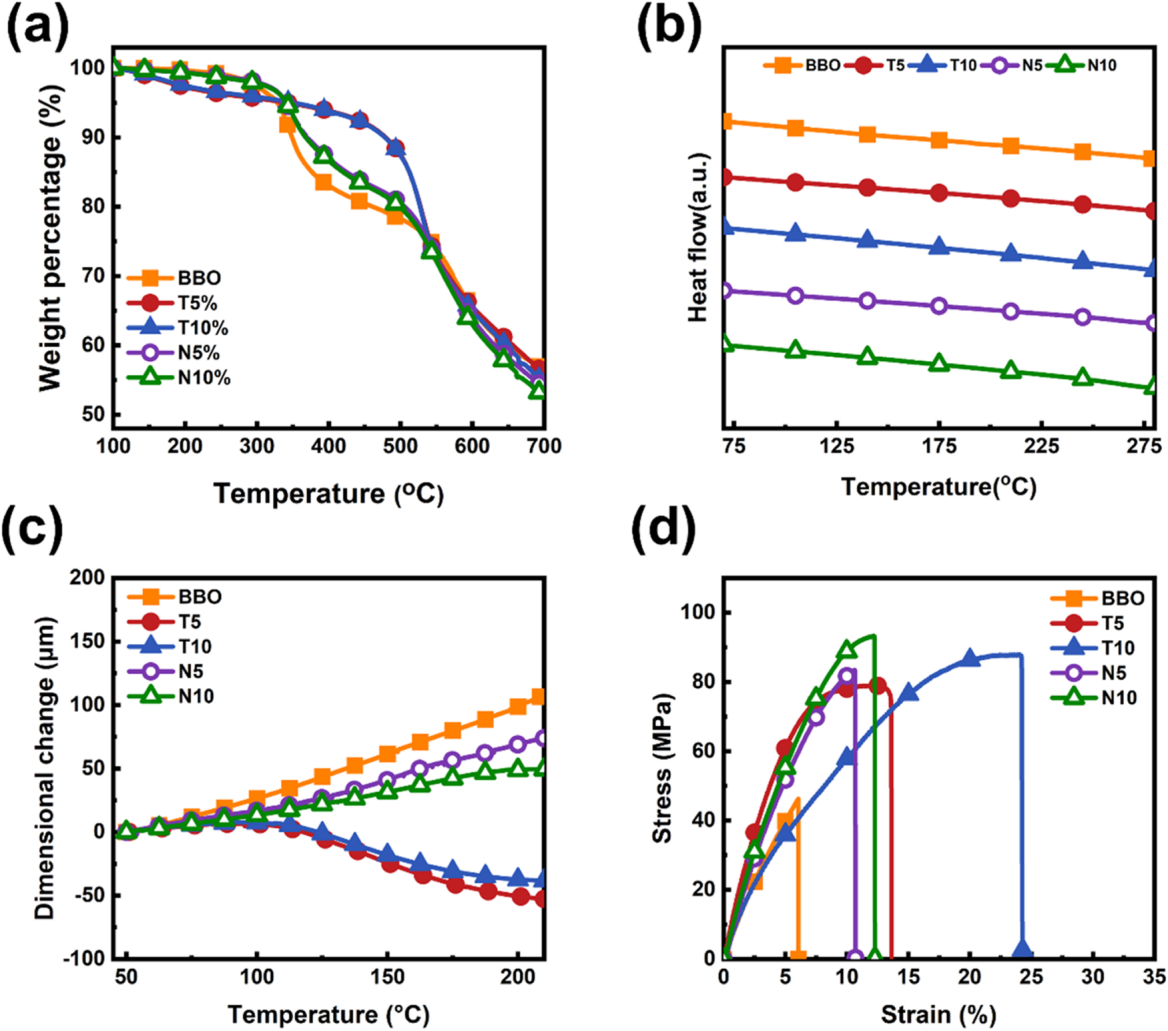
Thermal and mechanical properties of the SPI films: (a) TGA under a nitrogen atmosphere, (b) DSC at a ramping rate of 10 °C/min, (c) TMA at a ramping rate of 5 °C/min, and (d) stress–strain curves at a tensile rate of 3 mm/min.

The *T*_g_ of the PI samples was determined by using DSC, and the results are shown in Figure 2b. All samples exhibited *T*_g_ values above 280 °C, indicating highly rigid backbone structures and excellent thermal resistance. However, for some samples, *T*_g_ approached or even exceeded the thermal decomposition temperature of the polymer, rendering *T*_g_ undetectable by DSC. Despite this, the observed high thermal stability suggests that the polymer backbones were effectively designed to maintain dimensional stability at elevated temperatures. Even after incorporating bulky alicyclic structures, the samples retained high *T*_g_ values, indicating that the design successfully balances rigidity, processability, and thermal stability, thereby highlighting the potential for applications in high-temperature environments.

Regarding thermomechanical properties, the results of TMA analysis are presented in Figure 2c. The CTE values are presented in Table 1. The pristine BBO film exhibited relatively high structural regularity and partial crystallinity, resulting in thermal expansion behavior primarily dominated by its crystalline regions, which limited intermolecular hydrogen bonding. With the incorporation of alicyclic monomers, particularly in the NBDA series, the rigid and bulky alicyclic structures disrupted the orderly stacking of polymer chains, leading to the formation of block copolymer-like microphase-separated structures. This effectively reduced the CTE from 110 to 51 and 39 ppm/K for N5 and N10, respectively, with a clear downward trend as the alicyclic content increased. The introduced irregularity and steric hindrance suppressed the thermally induced chain motion, thereby enhancing the dimensional stability. Notably, the TCDDA series exhibited negative CTE because, during thermal imidization, the PI backbone aligned highly along the film plane (high in-plane orientation) under dipole–dipole and hydrogen bonding interactions, as shown in Figure 3. The tricyclodecane backbone of TCDDA, with significant steric hindrance and noncoplanar chain segments, inhibited close intermolecular packing and reduced crystallinity, resulting in a low-crystalline or nearly amorphous film. This structure, combined with high orientation, led to anisotropic thermal deformation upon heating: chain segments in the *xy-*direction were constrained by hydrogen bonding and orientation, suppressing expansion. In contrast, those in the *z-*direction were less restricted and expanded more. To maintain volumetric expansion balance, the pronounced *z*-direction expansion caused contraction in the *xy-*direction, ultimately producing a negative in-plane CTE.^33,35^ This behavior differed from that of the NBDA series. Overall, the introduction of alicyclic structures improved the thermal dimensional stability through a combination of increased structural freedom and an enhanced hydrogen bonding network.

**Figure 3:**
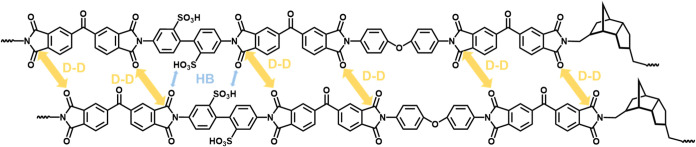
Mechanism of the in-plane chain alignment of TCDDA-based SPI induced by dipole–dipole (D–D) and hydrogen bonding (HB) interactions.

**Table 1:** Film Density, PAA Inherent Viscosity, Molecular Weight, Water Uptake, Swelling Ratio, and Thermal and Mechanical Properties of the SPIs

SPI	ρ (g/cm^3^)	η_inh_ (dL/g)	*M*_n_	*Đ*	*T*_d_^5%^ (°C)	*T*_g_ (°C)	CTE (ppm/K)	UTS (MPa)	ε_b_ (%)
BBO	1.359	0.52	39300	2.04	328	>280	110	64	10.5
T5	1.361	0.59	44500	2.08	342	>280	–29	80	12.2
T10	1.366	0.65	44700	1.99	351	>280	–22	89	22.7
N5	1.349	0.43	29800	2.50	340	>280	51	86	10.5
N10	1.346	0.44	34300	2.26	340	>280	39	92	12.3

The mechanical properties of the membranes were further evaluated, and the corresponding stress–strain curves are shown in Figure 2d. The mechanical parameters, including UTS and ε_b_, are presented in Table 1. The incorporation of alicyclic structures significantly enhanced the mechanical performance of all samples, highlighting the reinforcing effect of alicyclic monomers in molecular design. This enhancement is attributed to the rigid, noncoplanar alicyclic structures that impose substantial steric hindrance, effectively restricting chain mobility and improving deformation resistance. Additionally, the microstructural disorder introduced by these monomers helps dissipate localized stress, thereby enhancing the toughness. Among all tested samples, the NBDA series showed the most pronounced performance. Compared to the unmodified BBO matrix, the N5 and N10 films demonstrated markedly higher tensile strength while maintaining good elongation, indicating that proper incorporation of alicyclic structures can simultaneously increase stiffness and preserve ductility, endowing the material with high toughness and load-bearing capacity. For the TCDDA-based samples, although the original BBO backbone exhibited tightly ordered packing, the addition of alicyclic monomers induced local chain relaxation and decreased overall ordering. However, the increase in molecular mobility improved intermolecular interactions and energy dissipation capability, resulting in a more stable tensile behavior. Moreover, the TCDDA series exhibited relatively higher molecular weights, which contributed to better chain continuity and entanglement, thereby enhancing the overall mechanical strength and fracture toughness. Notably, the T10 sample demonstrated a high ε_b_ of 22.7%, exhibiting a ductile and durable mechanical response. Overall, the incorporation of alicyclic structures not only enhances rigidity and strength but also improves toughness by modulating chain mobility and introducing structural irregularities, thereby further reinforcing the structural stability and durability of SPI membranes in practical applications.

### Morphological Characterizations of the PI Thin Films

Microstructural analysis was conducted using SEM, AFM, and GIWAXS. As shown in Figure 4, SEM images revealed the formation of distinct phase-separated structures on the membrane surface after the introduction of alicyclic diamines. With increasing alicyclic content, the surface morphology became progressively rougher. This phase separation facilitates ion transport by promoting the aggregation of hydrophilic domains, while the hydrophobic segments contribute to maintaining the overall structural integrity. According to the AFM results, as shown in Figure S2, distinct bright and dark regions, corresponding to hydrophobic and hydrophilic areas, respectively, confirm the presence of a microphase-separated morphology in the membranes. In the TCDDA-based sample, the pronounced domain contrast and continuous distribution indicate well-developed phase separation with hydrophobic backbones forming elevated regions and sulfonated hydrophilic segments forming lower regions. Variations in domain size and surface roughness among the samples suggest that incorporating alicyclic diamines influences both the degree and scale of phase separation, consistent with the improved ion selectivity observed in the VRFB performance tests. In addition, Figure 5 shows that the GISAXS characteristic *q* peaks of BBO, T5, and T10 are 0.17, 0.09, and 0.11 nm^–1^, corresponding to domain spacings (*d* = 2π/*q*) of approximately 36.9, 69.8, and 57.1 nm, respectively. T5 exhibits the smallest *q* value and the largest domain spacing, further confirming the presence of a distinct and well-developed phase-separated structure in the membranes. To further investigate the internal microstructural arrangement, GIWAXS was employed to analyze samples with a film thickness of approximately 4 μm, focusing on the crystalline domains derived from the BDSA blocks. Both the 2D diffraction patterns and the integrated one-dimensional (1D) profiles are presented in Figure S3. The 1D profiles were obtained through full-range azimuthal integration, showing *q* values in the range of 1.55–1.59 Å^–1^, corresponding to *d*-spacings of 3.95–4.06 Å. Overall crystallinity decreased upon incorporation of alicyclic monomers. However, this reduction was less significant in samples containing TCDDA compared to those with NBDA. This can be attributed to the difference in the molecular geometry: TCDDA has a single, rigid tricyclic backbone that, at sufficient loading, introduces significant steric hindrance, as reflected by the increase in fractional free volume (FFV, shown in Figure S4) from 9.9% in BBO to 10.1% in T10. This increment highlights the minor compositional differences that effectively affect the microstructures. This steric effect disrupts tight chain packing while enhancing the driving force for phase separation between the hydrophilic and hydrophobic segments, leading to more distinct and stable domain distributions. In addition, the Brunauer–Emmett–Teller (BET) analysis of the T10 membrane has been conducted and is presented in Figure S5. However, the residual solvent blocked the pore areas, and the measured surface area negatively deviated. It is still evident from the MD simulations that TCDDA can enrich the free volume inside the SPI membrane. In contrast, NBDA contains multiple geometric isomers with more irregular three-dimensional conformations, resulting in greater irregular steric interference, looser packing, and a higher free volume. However, the excessive free volume and structural looseness in NBDA-based membranes reduce the stability of phase separation, increase water uptake and vanadium ion permeability, and thus lower ion selectivity and structural stability. For VRFB applications, the moderate FFV increase in TCDDA-based membranes facilitates the formation of tortuous and narrow proton transport channels, enabling efficient proton conduction while effectively blocking larger hydrated vanadium ions, thereby achieving a favorable balance between ion selectivity and structural stability. This aspect is evidenced in the following sections:

**Figure 4:**
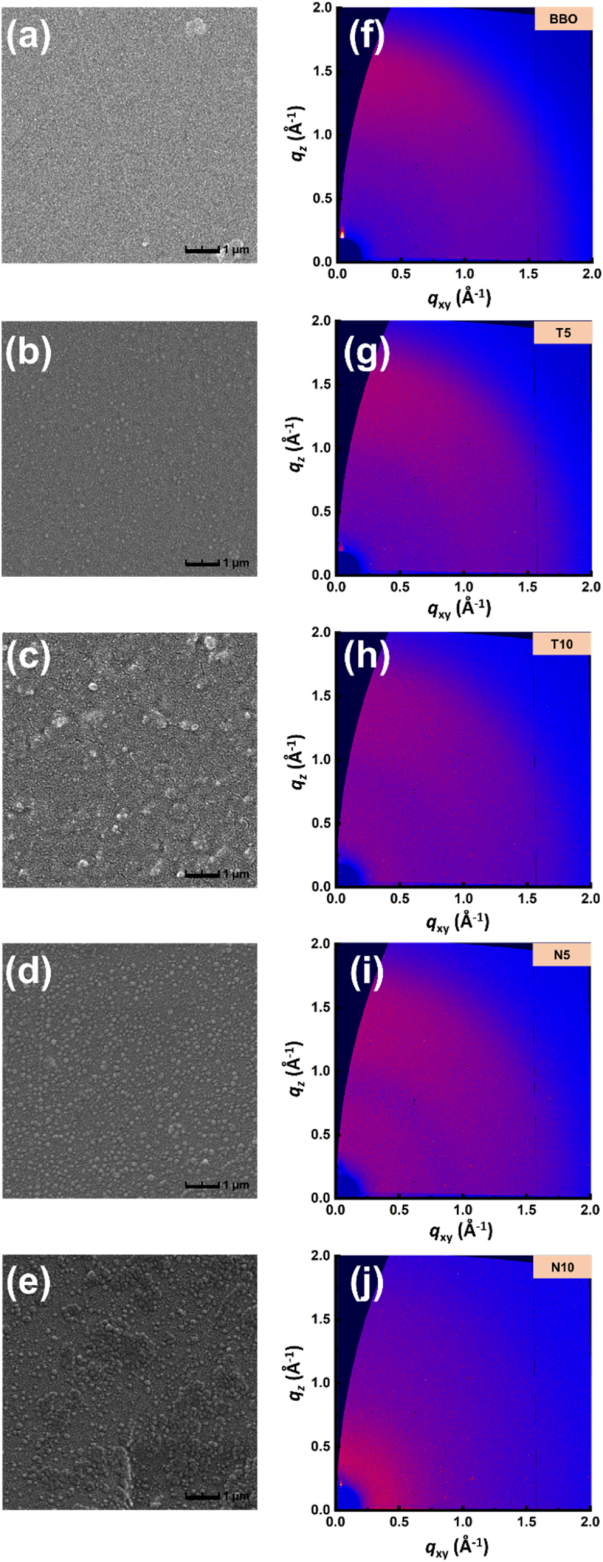
Surface morphology and microstructural analysis of the SPIs: (a–e) SEM images and (f–j) 2D GIWAXS patterns of the SPIs: (a, f) BBO, (b, g) T5, (c, h) T10, (d, i) N5, and (e, j) N10.

**Figure 5:**
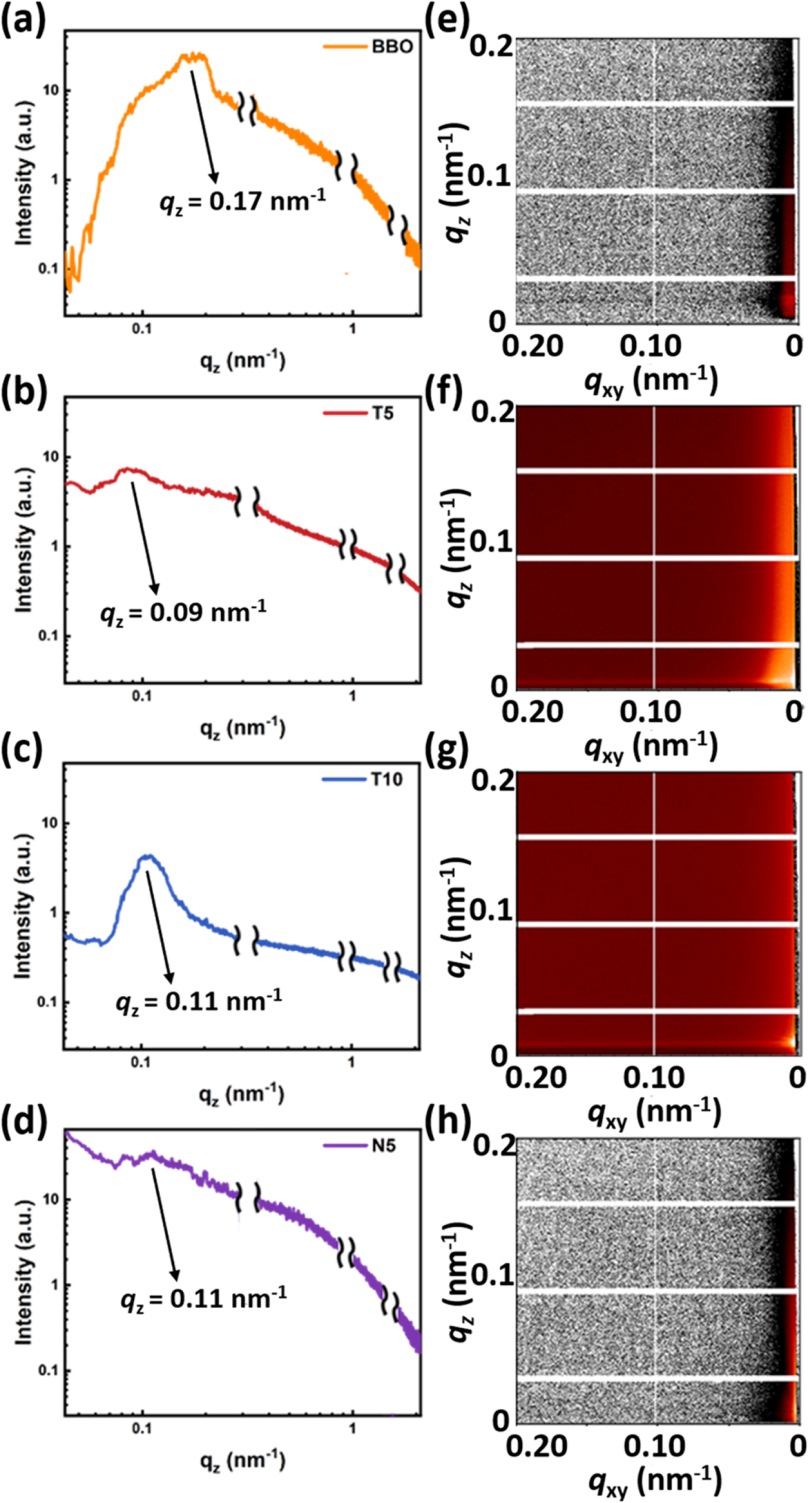
Microstructural analysis of the SPIs: (a–d) 1D GISAXS and (e–h) 2D GISAXS patterns of the SPIs: (a, e) BBO, (b, f) T5, (c, g) T10, and (d, h) N5.

### Ion Exchange and VRFB Performance of the SPI Films

The SPI’s stability test in water was conducted, and the corresponding parameters, including WU and SR, are presented in Table 2. The TCDDA series exhibited lower WU after the introduction of TCDDA-based alicyclic structures, likely due to the increased rigidity of their molecular structures. Specifically, the WU decreased from 6.8% in BBO to 4.7% in T10. In contrast, the NBDA series showed an upward trend, with N10 reaching 11.6%. In terms of SR, both series showed a decreasing trend, indicating that alicyclic structures help improve dimensional stability and regulate internal microporosity. For example, the SR_*xy*_ decreased from 5.06% in BBO to 2.53% in T10 and further to 1.27% in N10. Due to the hydrophobicity of alicyclic structures, the IEC of the TCDDA series slightly decreased from 1.196 mmol/g in BBO to 0.995 mmol/g in T10. In contrast, the NBDA series remained relatively unchanged, with N10 at 1.111 mmol/g. Notably, all SPI membranes exhibited IEC values higher than that of commercial Nafion 212 (IEC = 0.86 mmol/g), indicating a higher density of proton-donating sites. However, their proton conductivity remained much lower than that of Nafion 212, which reached 38.7 mS/cm. Regarding proton conductivity, the TCDDA series exhibited a slight decline, primarily attributed to its rigid structure, which increases density, reduces *d*-spacing, and promotes tight chain packing, thereby enhancing structural stability. Conversely, the NBDA series, with its more relaxed structure, larger *d*-spacing, and lower density, provided greater free volume and internal microporosity, facilitating water uptake and ion transport, thereby enhancing the conductivity. Overall, the introduction of TCDDA enhanced chain packing and structural stability. NBDA improved microporosity and ion accessibility, albeit with some compromise in water stability. However, the chain packing is a critical issue in blocking metal ion crossover, and these characteristics will be further investigated.

**Table 2:** Ion Exchange and Morphological Parameters of the SPI Films

polymer	WU (%)	SR_*xy*_ (%)	SR_*z*_ (%)	*q* (Å^–1^)	*d*-spacing (Å)	IEC (mmol/g)	σ (mS/cm)	*P* (cm^2^/min)
BBO	6.8	5.06	6.88	1.57	3.99	1.196	3.9	1.21 × 10^–7^
T5	6.3	4.94	5.93	1.58	3.96	1.098	3.2	1.25 × 10^–7^
T10	4.7	2.53	5.81	1.59	3.95	0.995	1.2	9.79 × 10^–8^
N5	9.2	3.75	4.84	1.57	3.99	1.123	7.1	1.04 × 10^–6^
N10	11.6	1.27	1.31	1.55	4.06	1.111	--^a^	2.65 × 10^–5^
nafion 212	--	--	--	--	--	0.860	38.7	3.14 × 10^–6^

a N10 ruptured during measurement, and its σ was not determined.

According to the vanadium ion permeability test shown in Figures 5a andS6, both the BBO and TCDDA series exhibit extremely low permeability, whereas the NBDA series shows significantly higher permeability. Notably, N10 even surpasses Nafion 212. Specifically, the T10 membrane showed a vanadium ion permeability of 9.79 × 10^–8^ cm^2^/min, significantly lower than that of 3.14 × 10^–6^ cm^2^/min for Nafion 212, representing nearly a 30-fold reduction. This superior barrier effect highlights the excellent vanadium-blocking ability of the TCDDA series. In contrast, the NBDA-based N10 membrane reached a permeability of 2.65 × 10^–5^ cm^2^/min, which is almost an order of magnitude higher than that of Nafion, indicating poor ion selectivity and a greater risk of vanadium accumulation and membrane degradation during VRFB operation.

To evaluate the chemical stability in the VRFB electrolyte, all PEMs were soaked in the VRFB electrolyte for 14 days before cycling. The results indicate that the BBO and NBDA series suffer from relatively poor stability; thus, the TCDDA series, with better structural integrity, was selected for actual VRFB testing. Figure 6b–d presents the battery rate performance results in terms of CE, VE, and EE. Cells assembled with T5, T10, and commercial Nafion membranes were tested under current densities ranging from 40 to 80 mA/cm^2^. As the current density increased, CE improved in all samples due to the shortened charge–discharge duration, which effectively suppressed vanadium ion crossover. Among them, T10 exhibited excellent CE under all tested conditions, attributed to its extremely low vanadium permeability. Conversely, VE decreased with increasing current density, primarily due to the rise in current flowing through the membrane and electrolyte, which resulted in a higher system resistance of the IR drop from ohmic polarization. Comparing T5 and T10, T5 showed slightly higher VE at 40 and 60 mA/cm^2^, possibly due to T10’s higher internal resistance or denser structure, which exacerbated polarization. As the product of CE and VE, the EE remained above 80% for all membranes at 40 mA/cm^2^. Although the EE slightly declined with increasing current density, T10 maintained excellent and stable performance at 60 and 80 mA/cm^2^. Its outstanding vanadium-blocking ability and high CE effectively offset the loss in VE, leading to performance comparable to or better than Nafion and T5. While T10’s proton conductivity remains lower than Nafion 212’s (38.7 mS/cm), its extremely low vanadium ion permeability enables stable and efficient output even under high-current operation. These results demonstrate that T10 possesses excellent ion selectivity and operational stability under demanding conditions, underscoring its potential as a high-performance membrane material.

**Figure 6:**
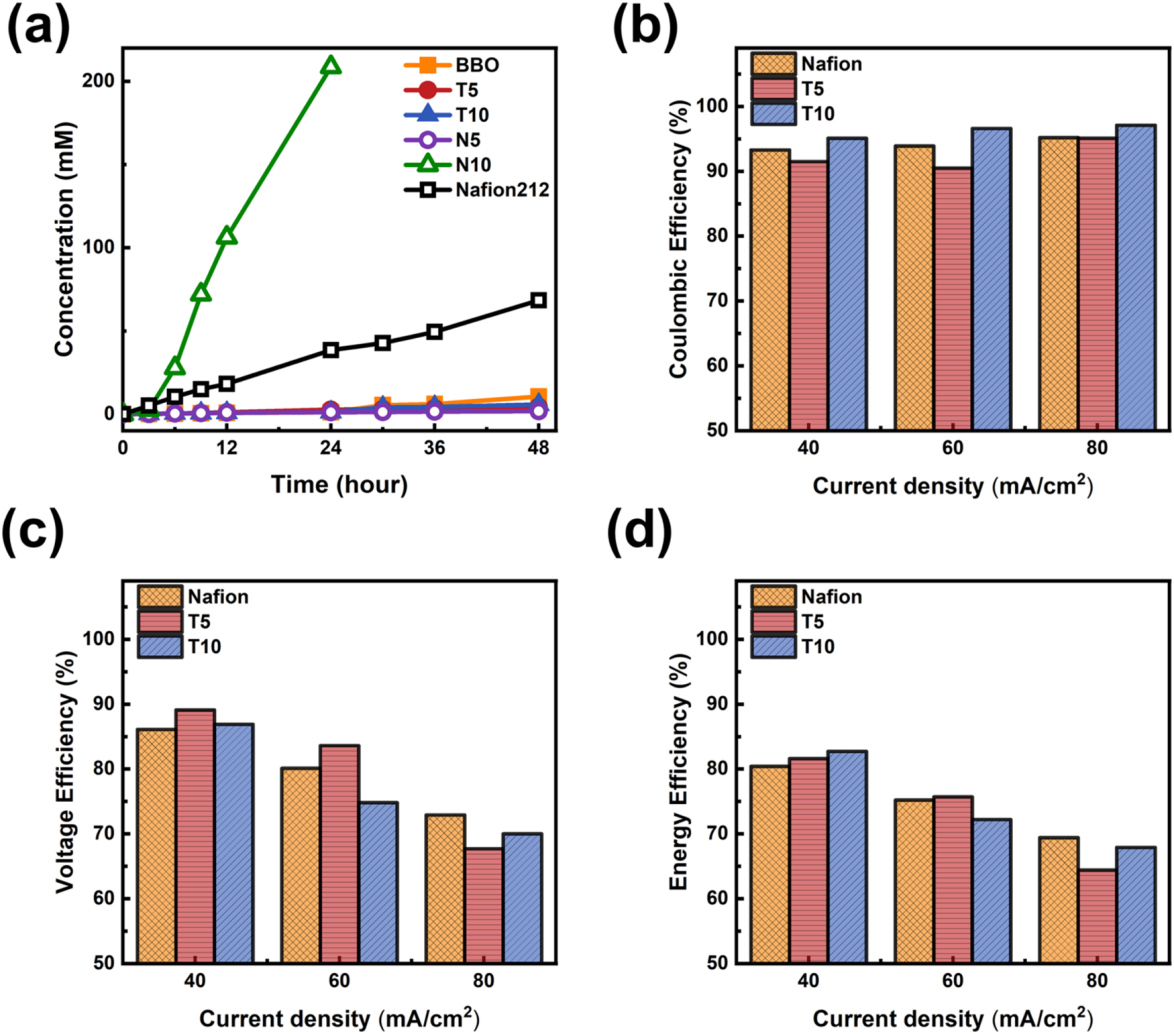
Ion exchange and electrochemical performance evaluations of the SPI membranes in VRFB systems: (a) vanadium ion permeability, (b) Coulombic efficiency (CE), (c) voltage efficiency (VE), and (d) energy efficiency (EE).

Subsequently, T10 was subjected to a long-term cycling test, as shown in Figure 7a. After 25 cycles at 40 mA/cm^2^, the cell maintained a good operational stability. Postcycling analysis was conducted via FTIR and SEM, as shown in Figure 7b,c. The FTIR spectra showed the disappearance of a characteristic peak near 1600 cm^–1^ after testing, possibly due to partial oxidative degradation of the aromatic moieties in the main chain. These characteristics highlight that film packing plays an essential role in blocking vanadium ion crossover and enhancing VRFB stability. SEM images also revealed the formation of additional microcracks on the membrane surface due to prolonged vanadium ion penetration and redox reactions, indicating structural deterioration over time. Nevertheless, the T10 membrane retained its structural integrity and conductivity after extended operation, demonstrating excellent chemical stability and potential for application. These results indicate that the T10 membrane holds substantial promise for use in advanced energy systems requiring high cycling durability and ion selectivity.

**Figure 7:**
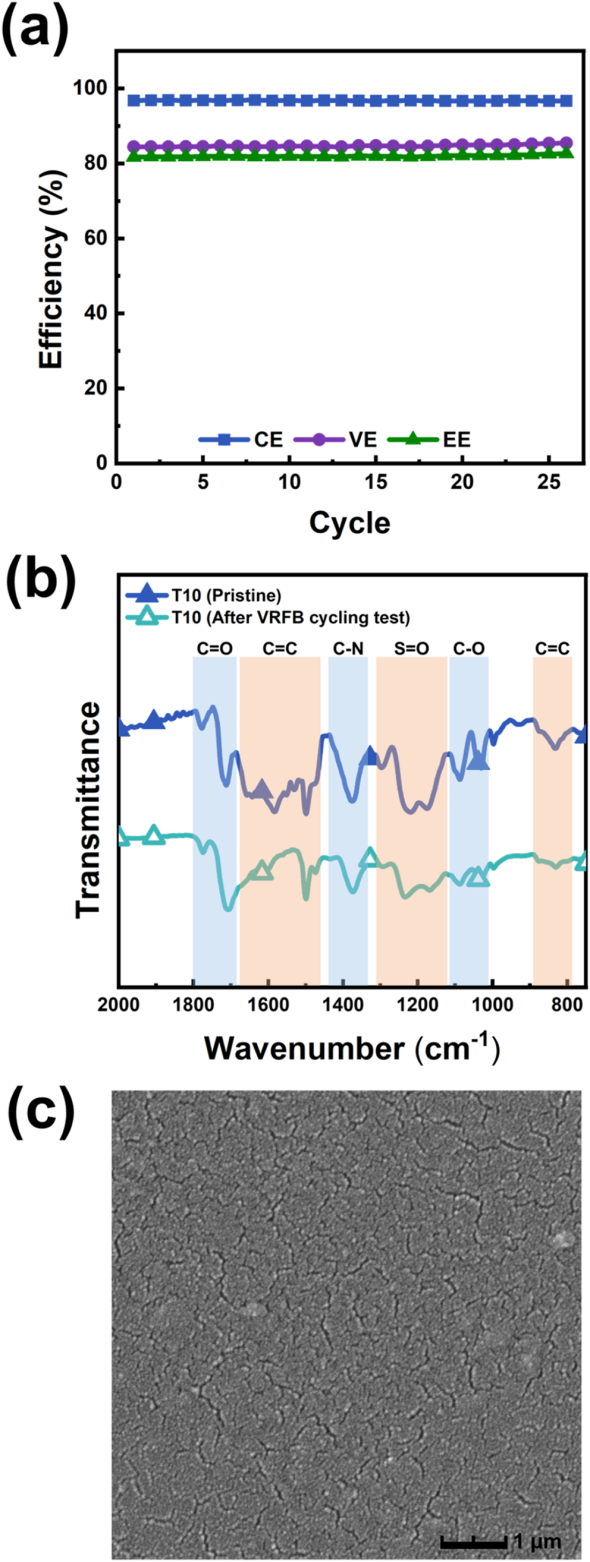
Cycle stability and ex-situ characterizations of the T10 membrane after the VRFB cycle test: (a) charge–discharge cycling performance, (b) FTIR spectra, and (c) SEM images of the cycled T10 membrane.

## Conclusions

This study successfully synthesized and systematically investigated SPI films with varying alicyclic content. By adopting a block copolymer strategy, TCDDA, a highly rigid and sterically hindered monomer, was introduced to modulate the polymer architecture. This design enables precise control over chain stacking distance, free volume, and phase separation without interfering with polymerization or film formation, thereby offering excellent dimensional stability and proton selectivity. All SPI membranes demonstrated outstanding thermal stability (*T*_d_^5%^ > 340 °C) and mechanical strength. Molecular weight analysis demonstrated that TCDDA-containing polymers possessed higher molecular weights, indicating improved polymerization efficiency likely due to reduced salt formation. The geometric structure and noncoplanarity of TCDDA effectively disrupted ordered chain packing, promoted stable microphase separation, and provided uniform pore distribution without sacrificing structural regularity. SEM and GIWAXS further revealed that TCDDA contributed to improved surface uniformity and internal structural stabilization, facilitating the formation of proton-conducting channels and reducing vanadium ion crossover while also showing minimal *d*-spacing variations that reflect a favorable balance between free volume and interchain interactions. In VRFB applications, the T10 membrane demonstrated an ultralow vanadium ion permeability and consistently excellent CE across current densities of 40–80 mA/cm^2^, reaching up to 98.6% and maintaining CE > 90% even under high-current conditions. Its EE reached 84.3% at 40 mA/cm^2^ and remained above 80% at 80 mA/cm^2^, outperforming both Nafion- and NBDA-based membranes. These results underscore its superior ion selectivity and operational stability. Long-term cycling tests further showed that T10 retained robust structural and electrochemical stability after several cycles. FTIR and SEM analyses confirmed the chemical integrity of the SPI membrane under acidic oxidative environments. In summary, the TCDDA-based molecular design strategy proposed in this study successfully integrates thermal stability, mechanical strength, proton conductivity, vanadium ion blocking ability, and microstructural integrity into SPI membranes, achieving efficient ion-exchange performance. In particular, its exceptionally low vanadium ion permeability effectively suppresses the crossover of vanadium ions in different oxidation states, helping to reduce self-discharge and improve electrolyte stability. The alicyclic design of SPI can enhance the cycle life and performance in VRFB applications.

## Supplementary Material



## Notes

Optical absorption spectra of the films collected in the vanadium ion permeability test (PDF)

## References

[1] Yao Z., Zhang Z., Hu M., Hou J., Wu L., Xu T. (2018). Perylene-based sulfonated aliphatic
polyimides for fuel cell applications:
Performance enhancement by stacking of polymer chains. J. Membr. Sci..

[2] Kumar A. G., Saha S., Tiwari B. R., Ghangrekar M. M., Das A., Mukherjee R., Banerjee S. (2020). Sulfonated co-poly­(ether imide)­s
with alkyne groups: Fabrication of crosslinked membranes and studies
on PEM properties including MFC performance. Polym. Eng. Sci..

[3] S R. R. R., Rashmi W., Khalid M., Wong W. Y., Priyanka J. (2020). Recent Progress
in the Development of Aromatic Polymer-Based Proton Exchange Membranes
for Fuel Cell Applications. Polymers.

[4] Shirvanian P., van Berkel F. (2020). Novel components
in Proton Exchange Membrane (PEM)
Water Electrolyzers (PEMWE): Status, challenges and future needs.
A mini review. Electrochem. Commun..

[5] Zhang K., Liang X., Wang L., Sun K., Wang Y., Xie Z., Wu Q., Bai X., Hamdy M. S., Chen H., Zou X. (2022). Status and perspectives
of key materials for PEM electrolyzer. Nano
Res. Energy.

[6] Li L., Nakajima H., Moriyama A., Ito K. (2023). Theoretical analysis
of the effect of boiling on the electrolysis voltage of a polymer
electrolyte membrane water electrolyzer (PEMWE). J. Power Sources.

[7] Li J., Liu S., He Z., Zhou Z. (2016). Semi-fluorinated sulfonated polyimide
membranes with enhanced proton selectivity and stability for vanadium
redox flow batteries. Electrochim. Acta.

[8] Chen Q., Ding L., Wang L., Yang H., Yu X. (2018). High Proton
Selectivity Sulfonated Polyimides Ion Exchange Membranes for Vanadium
Flow Batteries. Polymers.

[9] Pu Y., Zhu S., Wang P., Zhou Y., Yang P., Xuan S., Zhang Y., Zhang H. (2018). Novel branched sulfonated polyimide/molybdenum
disulfide nanosheets composite membrane for vanadium redox flow battery
application. Appl. Surf. Sci..

[10] Wang L., Yu L., Mu D., Yu L., Wang L., Xi J. (2018). Acid-base
membranes of imidazole-based sulfonated polyimides for vanadium flow
batteries. J. Membr. Sci..

[11] Mauritz K. A., Moore R. B. (2004). State of understanding
of Nafion. Chem. Rev..

[12] Awulachew S. S., Nigussa K. N. (2024). A study of the performance
of SPEEK electrolyte of
a novel PEMFC. Mater. Res. Express.

[13] Kamal M., Jaafar J., Khan A. A., Khan Z., Ismail A. F., Othman M. H. D., Rahman M. A., Aziz F., Rehman G. U. (2024). A Critical
Review of the Advancement Approach and Strategy in SPEEK-Based Polymer
Electrolyte Membrane for Hydrogen Fuel Cell Application. Energy Fuels.

[14] Ryu S. K., Kim A. R., Vinothkannan M., Lee K. H., Chu J. Y., Yoo D. J. (2021). Enhancing Physicochemical
Properties and Single Cell
Performance of Sulfonated Poly­(arylene ether) (SPAE) Membrane by Incorporation
of Phosphotungstic Acid and Graphene Oxide: A Potential Electrolyte
for Proton Exchange Membrane Fuel Cells. Polymers.

[15] Ye Z., Chen N., Zheng Z., Xiong L., Chen D. (2023). Preparation
of Sulfonated Poly­(arylene ether)/SiO_2_ Composite Membranes
with Enhanced Proton Selectivity for Vanadium Redox Flow Batteries. Molecules.

[16] Mandal A. K., Bisoi S., Banerjee S. (2019). Effect of Phosphaphenanthrene
Skeleton
in Sulfonated Polyimides for Proton Exchange Membrane Application. ACS Appl. Polym. Mater..

[17] Yang P., Long J., Xuan S., Wang Y., Zhang Y., Li J., Zhang H. (2019). Branched sulfonated
polyimide membrane with ionic cross-linking
for vanadium redox flow battery application. J. Power Sources.

[18] Yu L., Wang L., Yu L., Mu D., Wang L., Xi J. (2019). Aliphatic/aromatic sulfonated polyimide
membranes with cross-linked
structures for vanadium flow batteries. J. Membr.
Sci..

[19] Zhang Y., Li J., Zhang H., Zhang S., Huang X. (2014). Sulfonated polyimide
membranes with different non-sulfonated diamines for vanadium redox
battery applications. Electrochim. Acta.

[20] Cao L., Kong L., Kong L., Zhang X., Shi H. (2015). Novel sulfonated
polyimide/zwitterionic polymer-functionalized graphene oxide hybrid
membranes for vanadium redox flow battery. J.
Power Sources.

[21] Li J., Yuan X., Liu S., He Z., Zhou Z., Li A. (2017). A Low-Cost and High-Performance Sulfonated
Polyimide Proton-Conductive
Membrane for Vanadium Redox Flow/Static Batteries. ACS Appl. Mater. Interfaces.

[22] Li J., Liu J., Xu W., Long J., Huang W., Zhang Y., Chu L. (2022). Highly ion-selective
sulfonated polyimide membranes with covalent
self-crosslinking and branching structures for vanadium redox flow
battery. Chem. Eng. J..

[23] Xu W., Long J., Liu J., Luo H., Duan H., Zhang Y., Li J., Qi X., Chu L. (2022). A novel porous
polyimide membrane with ultrahigh chemical stability for application
in vanadium redox flow battery. Chem. Eng. J..

[24] Chu J., Liu Q., Ji W., Li J., Ma X. (2023). Novel microporous sulfonated
polyimide membranes with high energy efficiency under low ion exchange
capacity for all vanadium flow battery. Electrochim.
Acta.

[25] Mi M.-C., Szu F.-E., Cheng Y.-C., Tsai C.-H., Chen J.-H., Huang J.-H., Kuo C.-C., Lin Y.-C., Leung M.-k., Chen W.-C. (2024). Semiaromatic Poly­(ester
imide) Copolymers with Alicyclic
Diamines for Low-K Properties at a High Frequency of 10–40
GHz. ACS Appl. Polym. Mater..

[26] Woo Y., Oh S. Y., Kang Y. S., Jung B. (2003). Synthesis and characterization
of sulfonated polyimide membranes for direct methanol fuel cell. J. Membr. Sci..

[27] Fuchs S., Lichte D., Dittmar M., Meier G., Strutz H., Behr A., Vorholt A. J. (2017). Tertiary
Amines as Ligands in a Four-Step
Tandem Reaction of Hydroformylation and Hydrogenation: An Alternative
Route to Industrial Diol Monomers. ChemCatChem.

[28] Fuchs S., Lichte D., Jolmes T., Rösler T., Meier G., Strutz H., Behr A., J Vorholt A. (2018). Synthesis
of Industrial Primary Diamines via Intermediate Diols–Combining
Hydroformylation, Hydrogenation and Amination. ChemCatChem.

[29] Choi C., Kim S., Kim R., Choi Y., Kim S., Jung H.-y., Yang J. H., Kim H.-T. (2017). A review of vanadium electrolytes
for vanadium redox flow batteries. Renew. Sustainable
Energy Rev..

[30] Li Z., He H., Zhai L., Guo H., Li X., Li T., He S., Chai S., Usenko A., Li H. (2025). Nafion Hybrid Membranes
with Enhanced Ion Selectivity via Supramolecular Complexation for
Vanadium Redox Flow Batteries. ACS Appl. Polym.
Mater..

[31] Li J., Xu F., Chen W., Han Y., Lin B. (2023). Anion Exchange Membranes
Based on Bis-Imidazolium and Imidazolium-Functionalized Poly­(phenylene
oxide) for Vanadium Redox Flow Battery Applications. ACS Omega.

[32] Hasegawa M., Horiuchi M., Wada Y. (2007). Polyimides
containing trans-1, 4-cyclohexane
unit (II). Low-K and Low-CTE semi-and wholly cycloaliphatic polyimides. High Perform. Polym..

[33] Hasegawa M. (2017). Development
of Solution-Processable, Optically Transparent Polyimides with Ultra-Low
Linear Coefficients of Thermal Expansion. Polymers.

[34] Cheng Y.-C., Chen Y.-C., Lin Y.-C., Kuo C.-C., Chen W.-C. (2023). Exploring
the Cross-Linking Effect on Decreasing the Dielectric Constant and
Dissipation Factor of Poly­(ester imide)­s at a High Frequency of 10–40
GHz. ACS Appl. Polym. Mater..

[35] Ishii J., Takata A., Oami Y., Yokota R., Vladimirov L., Hasegawa M. (2010). Spontaneous molecular
orientation of polyimides induced
by thermal imidization (6). Mechanism of negative in-plane CTE generation
in non-stretched polyimide films. Eur. Polym.
J..

