# Supplementary material for: Alicyclic Design of Sulfonated Polyimide Membranes with a Tricyclodecane Diamine for Improved Ion Crossover Blocking in Vanadium Redox Flow Batteries
Source: ACS Polym Au. 2025 Aug 25;5(5):589–602. doi: 10.1021/acspolymersau.5c00066 (PMC12511967; doi:10.1021/acspolymersau.5c00066)
Supplement: Supplementary file 1 [file lg5c00066_si_001.pdf]

Supporting Information

Alicyclic Design of Sulfonated Polyimide Membranes with A Tricyclodecane Diamine for Improved Ion Crossover-Blocking in Vanadium Redox Flow Batteries

Chieh-Yuan Chang,[a] *Chang-Liang Liu,*[a] *Shi-Jie Wang,*[a] *Fu-En Szu,*[b]

Hong-Yu Lin,[c] *Kao-Shu Chuang,*[c] *Man-kit Leung,*[b,d] *Yan-Cheng Lin,*[a,d*]

[a] Department of Chemical Engineering, National Cheng Kung University, Tainan 70101, Taiwan

[b] Department of Chemistry, National Taiwan University, Taipei 10617, Taiwan.

[c] Department of Green Material Technology, Green Technology Research Institute, CPC Corporation, Kaohsiung City 811, Taiwan

[d] Advanced Research Center for Green Materials Science and Technology, National Taiwan University, Taipei 10617, Taiwan.

[*] To whom all correspondence should be addressed: Y. C. Lin (E-mail: ycl@gs.ncku.edu.tw)

Scheme S1. Synthesis of the BDSA-Et_3NH

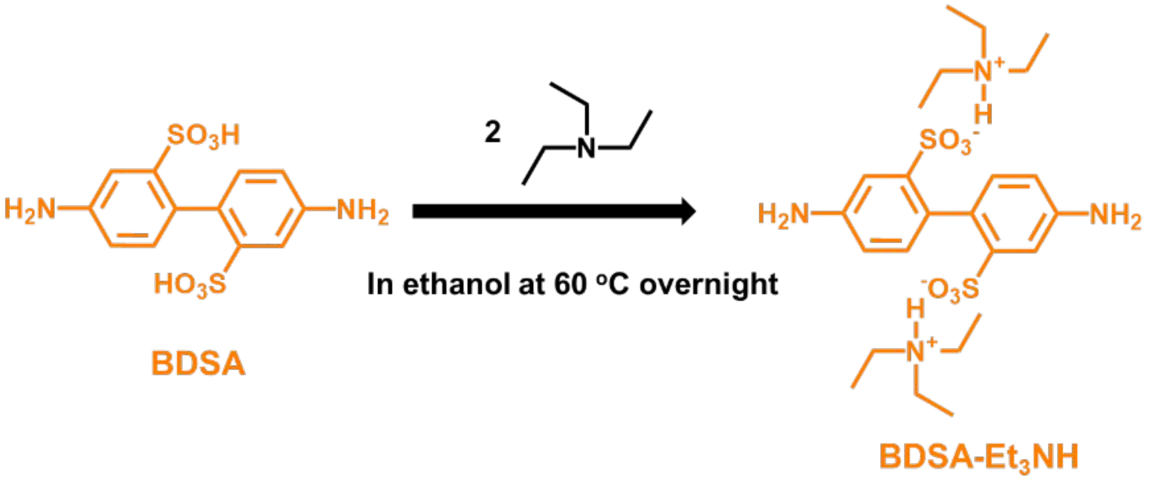

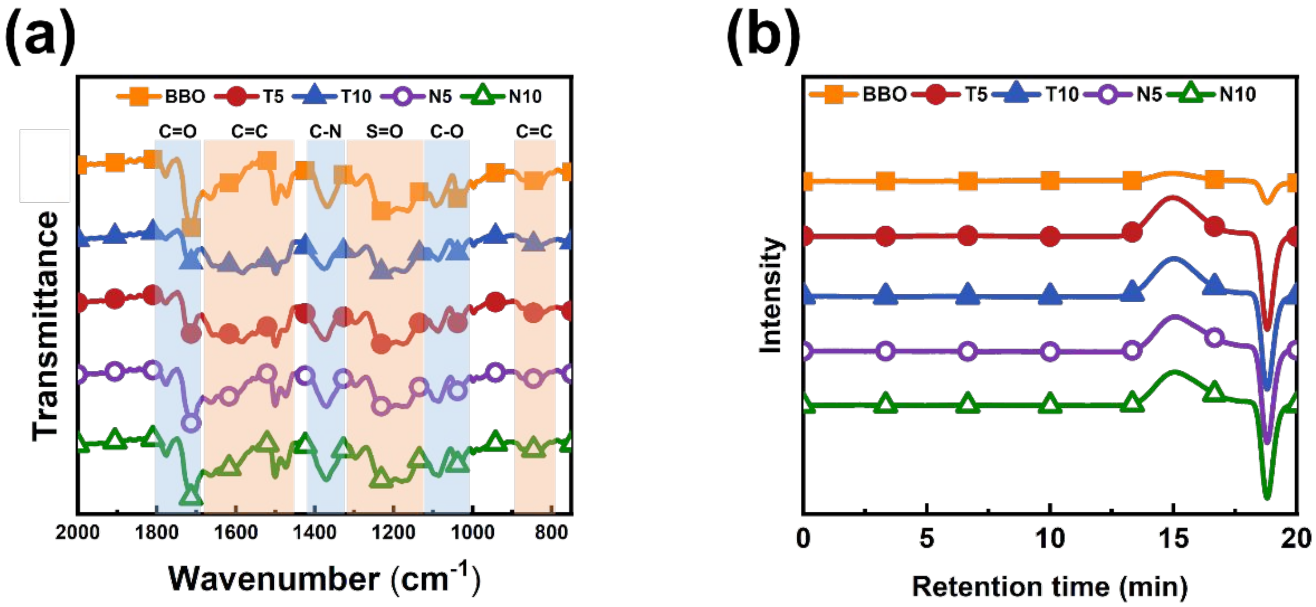

Figure S1. Chemical structure characterization of the SPIs: (a) FTIR spectra and (b) SEC analysis of molecular weight distribution.

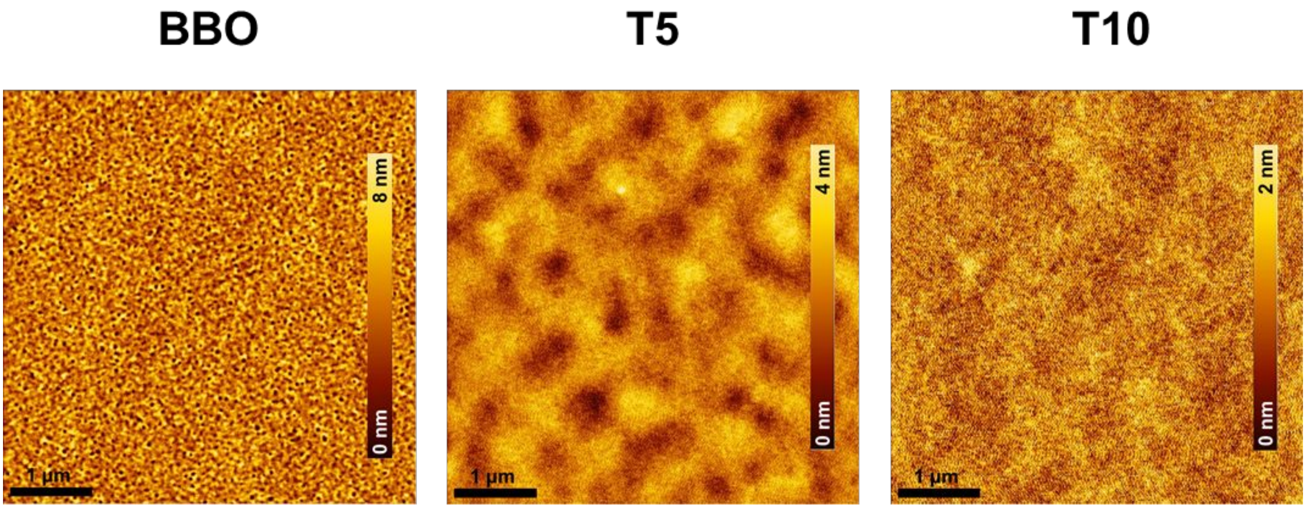

Figure S2. AFM height images (1 μm × 1 μm) of the SPI membranes: BBO (left), T5 (middle), and T10 (right).

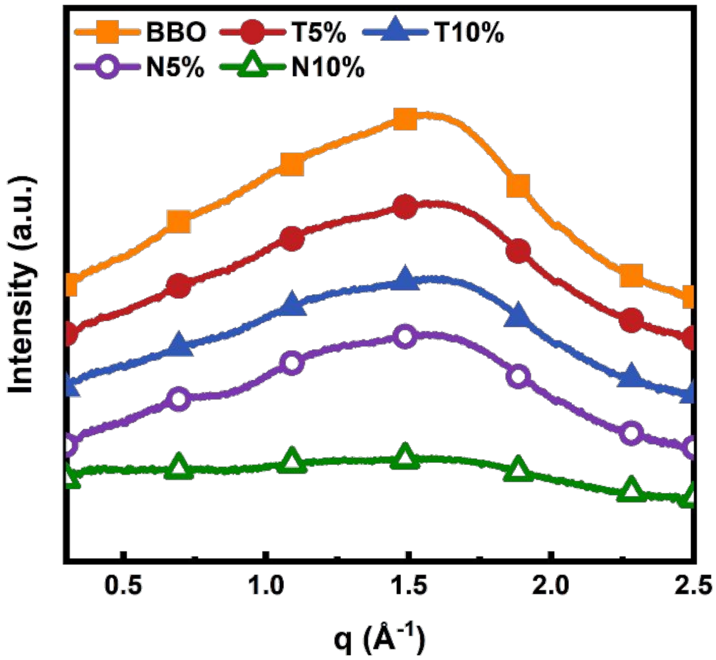

Figure S3. 1D GIWAXS line-cutting profiles of the SPI thin films.

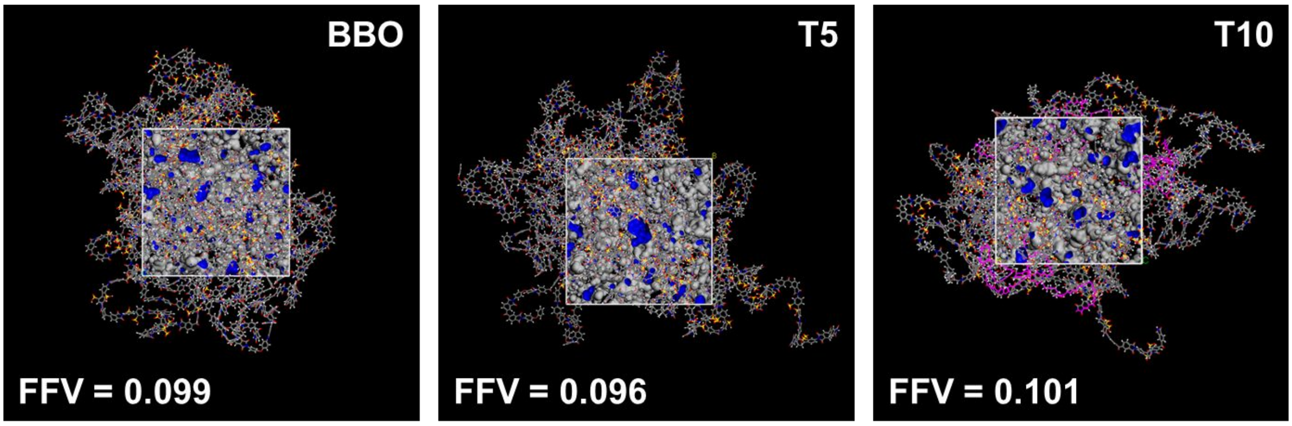

Figure S4. MD simulation results for free volume fraction (FFV) calculations. The simulation method was modified from a reported method for polyimides: Lei, H.; Li, X.; Wang, J.; Song, Y.; Tian, G.; Huang, M.; Wu, D. *Chem. Phys. Let.* **2022**, *786*, 139131. The MD simulations were conducted using the COMPASS II force field from the Forcite module in Materials Studio as follows: (i) A 40-ps NPT was performed at a high pressure of 0.5 GPa to compress the system. (ii) A 100-ps NPT was performed at 0.0001 GPa to relax the cell. (iii) The system was annealed from 298 to 598 K for 5 cycles using a 300-ps NPT at 0.0001 GPa. (iv) A second 100-ps NPT was performed at 0.0001 GPa was used to monitor density fluctuations. (v) A 200 ps NVT was performed to equilibrate the energy and obtain the final configuration—a total of 10 PI chains with a block architecture and 20 repeating units. For T5 and T10, there are 1 and 2 TCDDA units in a PI chain.

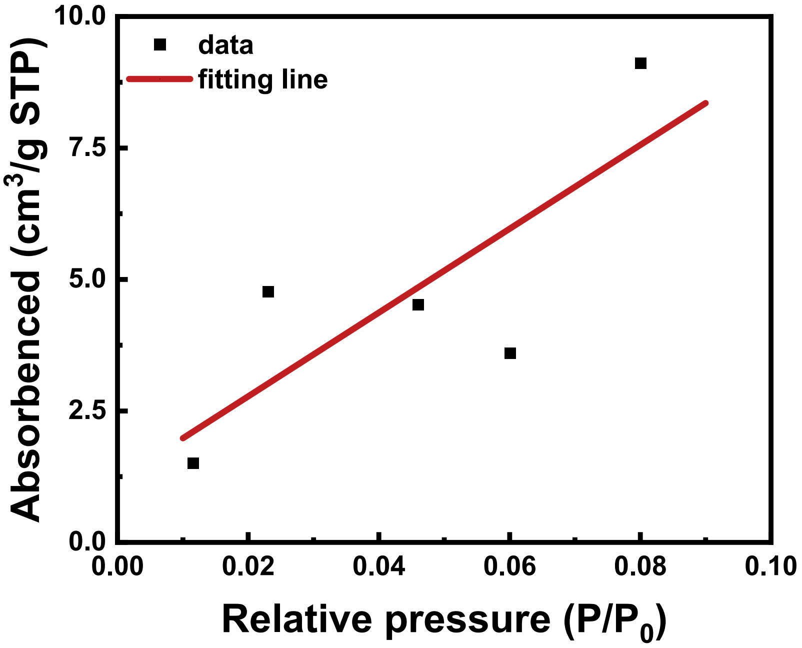

Figure S5. BET plot of T10 for specific surface area determination. Based on the Brunauer-Emmett-Teller (BET) test results (as shown in the linear fitting in **Figure S6**), the slope of the fitting line was 79.65 cm^3/g, and the intercept was 1.18 cm^3/g. According to the BET theory, the specific surface area was calculated to be 0.054 m^2/g, which is extremely small. This is likely because the strong interactions between the polymer and the solvent made it difficult to completely remove the solvent during thermal imidization and subsequent film detachment, thereby underestimating the specific surface area. Nevertheless, this result still reflects the relatively low water uptake of T10. There was a significant deviation from the fitting line beyond the relative pressure of 0.1. Therefore, the linear fitting only applies to the starting 5 points due to the negligible surface area of the membrane with a mass loading of 0.17 g.

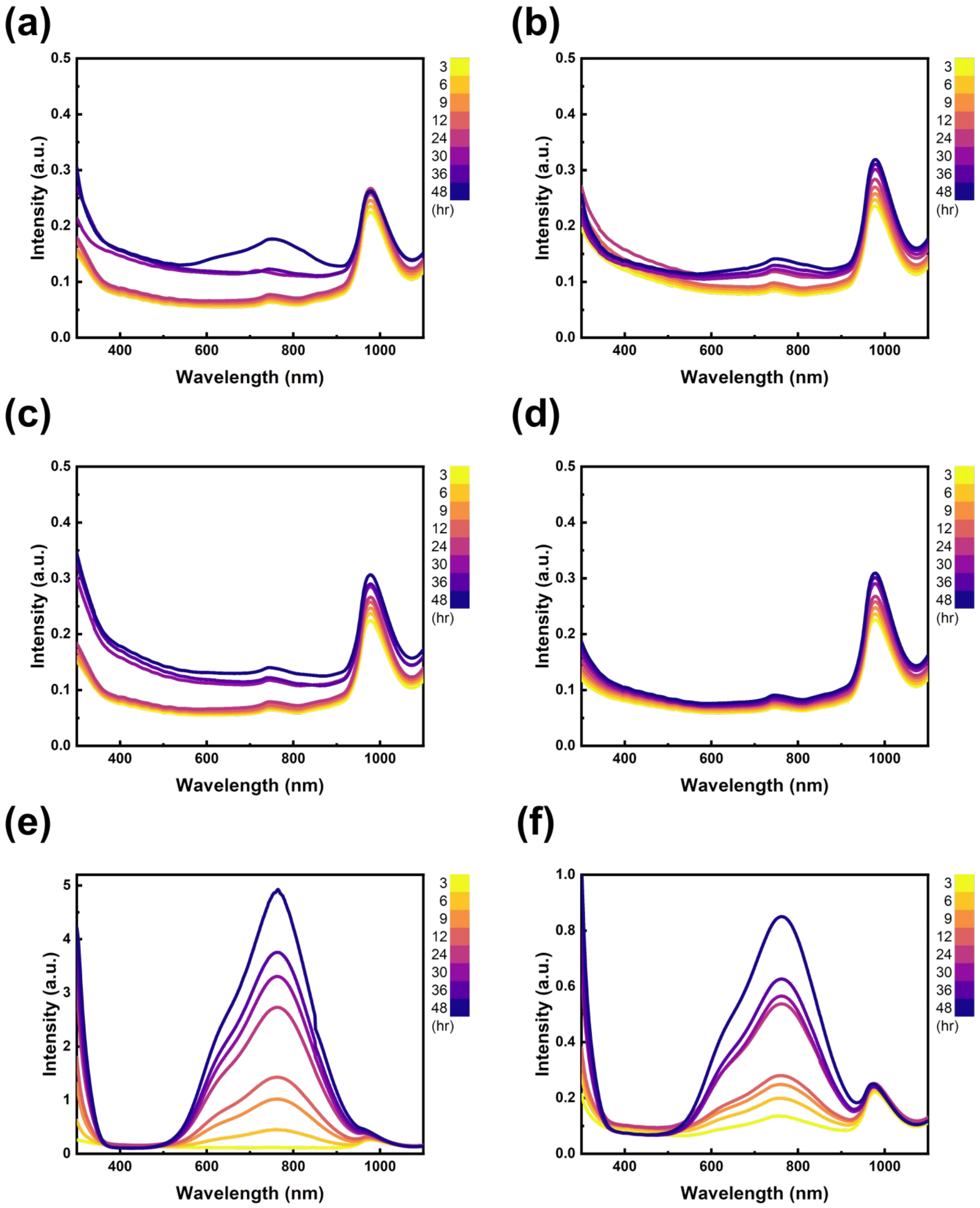

Figure S6. UV–vis absorption spectra of (a) BBO, (b) N5, (c) N10, (d) T5, (e) T10 and (f) Nafion 212 membranes in the vanadium ion permeability test.
